# Pseudomonas Aeruginosa Lung Infection Subverts Lymphocytic Responses through IL-23 and IL-22 Post-Transcriptional Regulation

**DOI:** 10.3390/ijms23158427

**Published:** 2022-07-29

**Authors:** Bérengère Villeret, Reem Ghinnagow, Saadé Kheir, Maëlys Born-Bony, Jay K. Kolls, Ignacio Garcia-Verdugo, Jean-Michel Sallenave

**Affiliations:** 1 Laboratoire d’Excellence Inflamex, Institut National de la Santé et de la Recherche Medicale U1152, Physiopathologie et Épidémiologie des Maladies Respiratoires, Université de Paris-Cité, 75006 Paris, France; berengere.villeret@inserm.fr (B.V.); reemghinajo@gmail.com (R.G.); saade.kheir@inserm.fr (S.K.); maelys.born-bony@inserm.fr (M.B.-B.); ignacio.garcia-verdugo@inserm.fr (I.G.-V.); 2 Center for Translational Research in Infection and Inflammation, Tulane School of Medicine, New Orleans, LA 70112, USA; jkolls1@tulane.edu

**Keywords:** IL-23, IL-17, lung, *Pseudomonas aeruginosa*, immunity, inflammation, cystic fibrosis, inflammation

## Abstract

Pseudomonas aeruginosa (*P.a*) is a pathogen causing significant morbidity and mortality, particularly in hospital patients undergoing ventilation and in individuals with cystic fibrosis. Although we and others have investigated mechanisms used by *P.a* to subvert innate immunity, relatively less is known about the potential strategies used by this bacterium to fight the adaptive immune system and, in particular, T cells. Here, using RAG KO (devoid of ‘classical’ αβ and γδ TCR T lymphocytes) and double RAG γC KO mice (devoid of T, NK and ILC cells), we demonstrate that the lymphocytic compartment is important to combat *P.a* (PAO1 strain). Indeed, we show that PAO1 load was increased in double RAG γC KO mice. In addition, we show that PAO1 down-regulates IL-23 and IL-22 protein accumulation in the lungs of infected mice while up-regulating their RNA production, thereby pointing towards a specific post-transcriptional regulatory mechanism not affecting other inflammatory mediators. Finally, we demonstrate that an adenovirus-mediated over-expression of IL-1, IL-23 and IL-7 induced lung neutrophil and lymphocytic influx and rescued mice against *P.a*-induced lethality in all WT, RAG γC KO and RAG γC KO RAG-deficient mice, suggesting that this regimen might be of value in ‘locally immunosuppressed’ individuals such as cystic fibrosis patients.

## 1. Introduction

Pseudomonas aeruginosa (*P.a*) is a pathogen causing significant morbidity and mortality, particularly in hospital patients undergoing ventilation and in patients with cystic fibrosis (CF) [1,2]. We and others have investigated the mechanisms used by *P.a* to subvert innate immunity. In particular, LasB, an important virulence factor with metalloprotease activity, has been shown to hamper lung defenses by targeting lung macrophages and epithelial cell responses, e.g., [3,4,5,6,7,8]. In addition, innate host mediators, such as neutrophil elastase (NE), have also been shown to be instrumental in down-regulating innate immune responses, including phagocytic and TLR receptors [1,2]. By contrast, relatively less is known about the potential strategies used by this bacterium to fight the adaptive immune system and, in particular, T cells.

Indeed, since ‘classical’ TCRαβ adaptive T cells [9,10], T cells with innate-like activities (TCRγδ, NKT cells, MAIT cells, [11,12,13,14,15,16]) or innate lymphoid cells [10] activated by non-cognate stimuli (NK cells, ILCs), have been shown to be able to control extra-cellular Gram bacteria (among which *P.a.*), it stands to reason that these pathogens may have, conversely, developed mechanisms to counteract the lymphocytic defensive armamentarium. Here, using RAG (recombination activated gene) KO (devoid of ‘classical’ αβ and γδ TCR T lymphocytes) and double RAG γC KO (devoid of both classical T, NK and ILC cells), we demonstrate that the lymphocytic compartment is indeed important to combat *P.a.* Recently [3], we showed that in vitro *P.a* infection of MPI (a macrophage cell line) targets the p40 chain common to IL-23 and IL-12 cytokines (both important cytokines known to prime Th17 and Th1 pathways, respectively). In keeping with this, we show here that *P.a* down-regulates IL-23 and IL-22 protein accumulation in the lungs of infected mice while up-regulating their RNA production, thereby pointing towards a specific post-transcriptional regulatory mechanism not affecting other inflammatory mediators. Finally, we demonstrate that an adenovirus-mediated over-expression of IL-1, IL-23 and IL-7 rescued mice against *P.a*-induced lethality in both WT and RAG-deficient mice.

Although cystic fibrosis patients are not bona fide immune-suppressed individuals, their deficiency in clearing lung pathogens such as *P.a* clearly indicates that new strategies are welcome to supplement already successful CFTR-targeted treatments. We, therefore, suggest that innovative therapies such as local supplementation of ‘immune-boosting’ cytokines such as those described in our ‘adenovirus regimen’ might be of value for these individuals.

## 2. Results

### 2.1. IL-17+, IL-22+ Tγδ and IL-17+ ILCs Increase Post Pseudomonas Aeruginosa Lung Infection, and Lymphocytes Are Important to Modulate Lung Resistance to Pseudomonas Aeruginosa Infections

**Figure 1:**
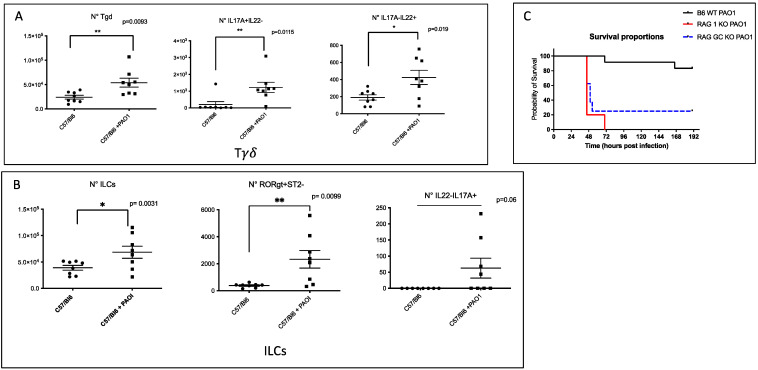
*P. aeruginosa* (PAO1) infection increases the levels of IL-17+ and IL 22+ Tγδ and ILCs. Male C57/Bl6 mice were infected for 16 h with *P. aeruginosa* (PAO1 strain, 10^7^ cfu = colony forming units). Animals were then culled, lungs isolated and treated for FACS analysis (see Materials and Methods, Figure S1 and Supplementary Table S1 for the list of antibodies used). Panel (**A**): shown are the number of total Tγδ cells, IL-17+ Tγδ, and IL-22+ Tγδ cells (see Figure S1 for the FACS gating strategy); Panel (**B**): shown are the number of total ILCs, ‘ILC3 ILCs’ (RORγt+), and IL-17 + ILCs (see Figure S1 for the FACS gating strategy). Each symbol represents an individual mouse. Unpaired t-tests have been performed to assess statistical significance (* *p* < 0.05, ** *p* < 0.01); Panel (**C**): C57/Bl6 WT (n = 12), RAG KO (*n* = 5) and RAG γC double KO (*n* = 8) mice were infected intra-nasally with 5.10^7^ cfu and survival curves were plotted using Kaplan–Meier curves. Statistical tests were performed using the Log-rank (Mantel–Cox) tests: C57/Bl6 PAO1 versus RAG KO: *p* < 0.0001; C57/Bl6 PAO1 versus RAG γC double KO: *p* = 0.0010; RAG KO versus RAG γC double KO: no statistical significance.

FACS analysis was performed in the lungs of mice 16 h post *P.a* (PAO1) infection (see Figure S1 and Table S1 for the FACS strategy). The most salient results are presented in Figure 1: the total numbers of lung Tγδ cells and that of IL-17+, IL-22+ Tγδ producing cells were increased (Panel A), as were those of total ILC3 and IL17+ ILC3s (Panel B).

Demonstrating the importance of these cells, we showed that RAG KO (devoid of ‘classical’ αβ and γδ TCR T lymphocytes) and double RAG γC KO (devoid of both classical T, NK and ILC cells) were more sensitive to PAO1 infection, compared to C57/Bl6 control mice (Figure 1C).

### 2.2. Lung IL-23 and IL-22 Protein Accumulation Is Down-Regulated Post PAO1 -Infection, Revealing a Post-Transcriptional Regulation

**Figure 2:**
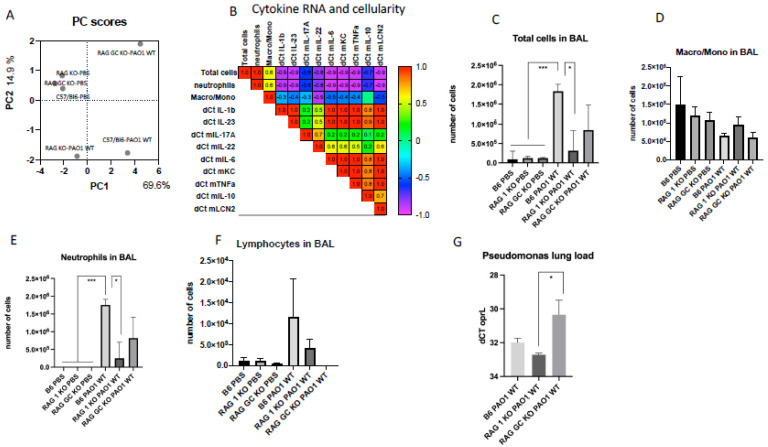
Cytokine RNA expression and lung inflammation post-*P. aeruginosa* infection. (**A**) C57/Bl6 WT, RAG KO and RAG γC double KO mice were infected intra-nasally with 1.4.10^7^ cfu of PAO1 (*n* = 5 in each group) or mock-treated with PBS (*n* = 3 in each group); 16 h later lungs were recovered for RNA assessment (RT-qPCR) and BAL performed for differential cell analysis (cytospins). An unbiased multivariate principal component analysis (PCA) global analysis, encompassing all 24 mice, was performed with the following variables: ‘total BAL cell numbers’, ‘total neutrophils’, total ‘monocytes/macrophages’, and IL-1b, KC, IL-17, IL-23, IL-6, IL-22, TNF, IL-10 lung RNA expression. (**B**) A correlation matrix was analyzed, plotting cytokine RNA levels (expressed as dCt. = Ct ‘mediator’-Ct 18S) and BAL cellularity. NB: the correlations between cytokine RNA levels and cells, although positive, appear *artefactually* negative (blue/purple color in the matrix) only because the value of dCT, the chosen RNA expression unit, is inversely proportional to gene expression. The numbers in the squares represent the r correlation value. (**C**–**F**) BAL cell cellularity is shown in each individual experimental group. (**G**) PAO1 load was assessed by RT-qPCR, using oprL (peptidoglycan associated protein) primers, a method previously validated (see Materials and Methods). NB: to reflect that dCT, the chosen RNA expression unit, is inversely proportional to gene expression, the units of the Y axis have been reversed for a more intuitive representation (dCT 34 to 28). Statistical significance was assessed with ANOVA test, followed by Tukey post hoc multivariate analysis. * *p* < 0.05; *** *p* < 0.005.

C57/Bl6, RAG KO and double RAG γC KO mice were then infected with a sub-lethal dose of PAO1 (or instilled with PBS as a Control), and lungs and BALs were collected 16 h later for BAL cell differential, RNA and ELISA analyses. We first performed, taking into account all six experimental groups (three PBS/*n* = 3 mice per group and three PAO1/*n* = 5 mice per group) an unbiased multivariate Principal component analysis (PCA) with the following variables: ‘total BAL cell numbers’, ‘total neutrophils’, total ‘monocytes/macrophages’, and IL-1b, KC, IL-17, IL-23, IL-6, IL-22, TNF and IL-10 lung RNA expression (Figure 2A). The three ‘PBS groups’ (C57/Bl6, RAG KO and RAG γc KO) clustered together, and the correlation matrix (Figure 2B) demonstrated that ‘neutrophils’ and ‘total cells’ were highly correlated. So was the RNA expression of most mediators, IL-1b, KC, IL-23, IL-6, TNF, Lcn-2 and IL-10 (red squares), with the notable exception of IL-17 and IL-22, which correlated with each other (r = 0.7), but not with the other mediators, suggesting a different activation pathway, likely due to their differential lymphoid cellular source. Additionally, ‘total cells’ and ‘BAL neutrophils’ were highly positively correlated with the ‘myeloid’ cytokines IL-1b, KC, IL-23, IL-6, TNF and IL-10 RNA expression (NB: although positive, that correlation appears *artefactually* negative (purple color in the matrix), only because the value of dCT, the chosen RNA expression unit, is inversely proportional to gene expression). As shown in Figure 2C–F, RAG and RAG γc KO mice had lower numbers of neutrophils and lymphocytes, in keeping with the notion that at that time point (16 h), lymphocytes partially direct the neutrophil influx in the lungs of infected mice [11]. Relatedly, reinforcing the lymphocyte–neutrophil axis, we showed that PAO1 clearance was less efficient in RAG γc KO mice, as evidenced by higher oprL *p.a* expression (Figure 2G).

**Figure 3:**
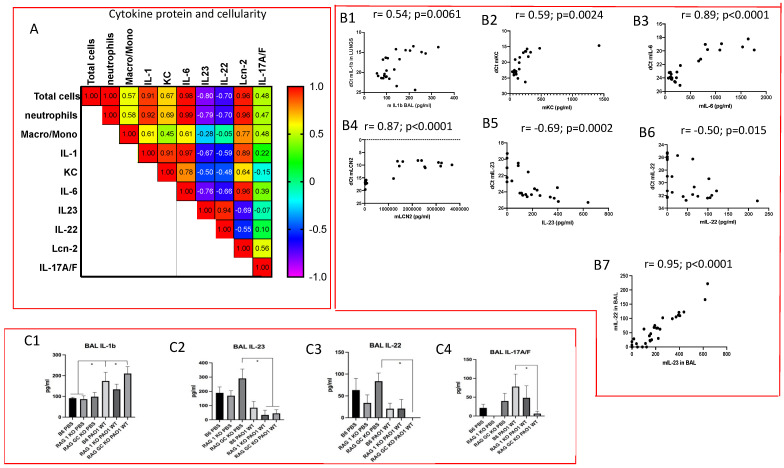
Cytokine protein expression reveals a post-transcriptional regulatory mechanism for IL-23 and IL-22 following *P. aeruginosa* infection. The same 6 groups (same 24 mice) as in Figure 2 were analyzed together, but at the cytokine protein levels (instead of RNA gene expression). (**A**) A correlation matrix was performed between cytokine protein levels (as assessed by ELISA) and between cytokine protein levels and BAL cellularity. (**B1**–**B7**) The positive and negative correlations between cytokine RNA and protein levels (**B1**–**B6**) and between IL-23 and IL-22 protein levels (**B7**) are plotted. All correlations (Pearson) and *p* values (two-tailed) were calculated with Prism 9. (**C1**–**C4**) BAL cytokine levels of IL-1b, IL-23, IL-22 and IL-17 are shown in each experimental group. Statistical significance was assessed with ANOVA test, followed by Tukey post hoc multivariate analysis. * = *p* < 0.05.

We then performed a similar analysis (Figure 3A) with the following variables: ‘total cell numbers’, neutrophils, and IL-1b, KC, IL-6, IL-23, IL-17, IL-22 and Lcn-2 BALF protein expression (instead of RNA expression as above). Notably, although IL-1b, KC, IL-6 and Lcn2 protein expression still correlated closely, and positively, to each other (Figure 3A, red/orange squares), akin to what was observed at the transcriptional level (see above Figure 2), IL-23, IL-17 and IL-22 clearly behaved differently. Notably, IL-23 and IL-22 protein levels correlated negatively with all other mediators (Figure 3A, blue squares) but positively with each other (Figure 3A, red square, and Figure 3B7).

Further confirming the specificity of IL-23/IL-22 expression, for each cytokine taken individually, IL-1, KC, IL-6, Lcn-2 RNA and protein levels correlated positively (Figure 3B1–B4), and IL-23 RNA, IL-22 RNA and their respective protein expression, by contrast, correlated negatively (Figure 3B5,B6). This suggested an important post-transcriptional regulation of IL-23 and IL-22 following PAO1 infection.

When the six different experimental groups were then split and compared with each other, the most salient results concerned IL-1b, IL-23, IL-17 and IL-22. Indeed, whereas IL-1b protein accumulation was not impacted by the absence of lymphocytes and was up-regulated by PAO1 infection (Figure 3C1), IL-23 and IL-22 were down-regulated by PAO1 treatment (Figure 3C2,C3). Furthermore, IL-17 and IL-22 levels were much less induced by PAO1 in RAG and RAG γc KO mice (IL-22 was indeed indetectable in RAG γc KO mice (Figure 3C3,C4), strengthening the notion that the latter two cytokines are mainly produced by lymphocytes during the infection, and at least in part (for IL-17) explaining the neutrophil influx at that time point (16 h).

**Figure 4:**
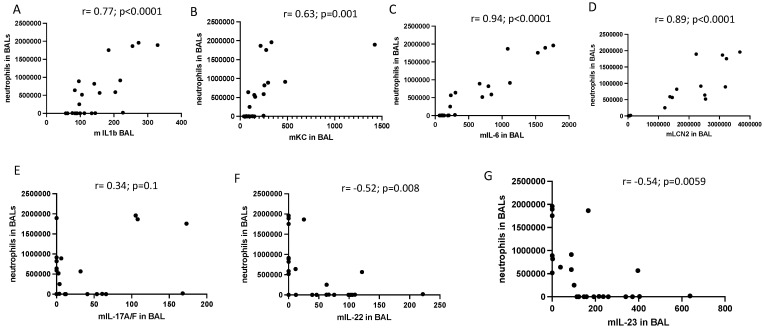
The BAL protein levels of IL-1b, KC, IL-6 and Lcn-2 correlate positively with BAL neutrophil influx, but not those of IL-23 and IL-22.

**Figure 5:**
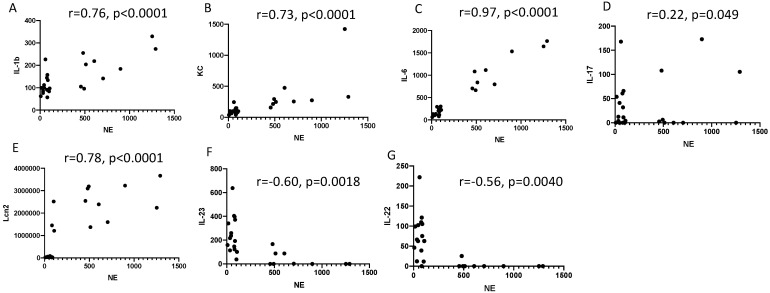
The BAL protein levels of IL-1b, KC, IL-6 and Lcn-2 correlate positively with neutrophil elastase activity, but not those of IL-23 and IL-22.

Correlations between the BAL protein levels of all mediators with neutrophils in PAO1 WT-infected mice were then specifically assessed and plotted in an X/Y format (Figure 4C). Of all the mediators (Figure 4A–G), IL-23 and IL-22 protein levels were the only ones inversely correlating with neutrophil influx (Panels F–G) and with neutrophil elastase proteolytic activity, which we used as an index of neutrophil activity (r= −0.60, *p* = 0.0018 and r = −0.56, *p* = 0.040, respectively, Figure 5F,G. This, and the uncoupling between IL-23 and IL-22 RNA and protein levels (see above), strongly suggested that this phenomenon was linked to neutrophilic inflammation, at least at this time point.

Correlations (Pearson, *p* values (two-tailed)) between BAL cytokine protein levels and neutrophil elastase (NE) activity (expressed in nM equivalent) from the same six groups (B6 PBS; RAG 1 KO PBS; RAG GC KO PBS; B6 PAO1 WT; RAG 1 KO PAO1 WT; RAG GC PAO1 WT, see Figure 2) are plotted.

**Figure 6:**
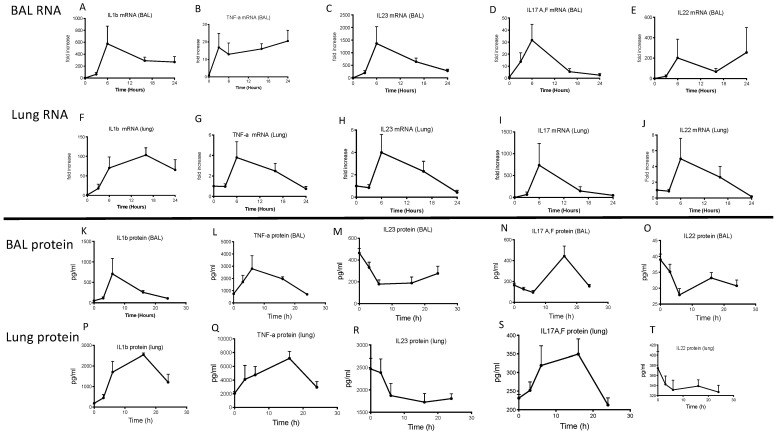
A kinetic study shows that IL-23 and IL-22 protein levels are down-regulated at an early time point following PAO1 lung infection.

Because the previous analysis had only been performed at a single time point (16 h), and because we and others have shown that neutrophils come as early as 3–4 h in PAO1 murine infections [11], we further analyzed the modulation of cytokine mRNA IL-23 and protein accumulation in an independent kinetic study studying both BAL and lung compartments, following PAO1 infection. These experiments demonstrated (Figure 6) differential effects of infection on cytokine levels, both in lung extracts and in BALF. At the RNA level (upper panels), all cytokines were induced at an early time point (3–6 h). In keeping with the described myeloid source of IL-1b, TNF and IL-23, the PAO1-mediated induction was much higher in BAL cells (mainly consisting of alveolar macrophages and neutrophils at 3–6 h) than in total lung cells.

By contrast, IL-17 mRNA was much more induced in lung cells (Panel 6I) than in BAL cells (Panel 6D), in keeping with the notion that IL-17 is mainly produced by ‘lung-residing cells’ such as innate or adaptive lymphocytes.

Relatedly, the protein accumulation of IL-17 in BALs (Panel N) and lungs (Panel S) occurred at a later time point (16 h onwards), again in accordance with the likelihood that T cells are responsible for this ‘late’ wave of IL-17 production [11]. Again, IL-23 and IL-22 mRNA and protein levels were shown to be uncoupled, both in BALF and lung extracts, with both proteins being down-regulated (panels M, R and O, T, respectively), and importantly, their reduction occurred at an early time point, coinciding with the influx of neutrophils.

Male C57/Bl6 WT mice were infected with 10^7^ cfu (*n* = 7 for each time point). At each time point, mice were culled, and their lungs were recovered. One lobe of the lung was homogenized in PBS in FastPrep-24 D tubes. After centrifugation, supernatants were recovered for ELISA analysis of cytokines. The other lobe was used for RNA preparation and cytokine gene expression. In parallel, BAL was performed, and after centrifugation, the cell pellet was used for RNA cytokine gene expression, while the supernatant was analyzed for cytokine protein content (ELISA). Each point represents the mean (*n* = 7) +/− SD.

**Figure 7:**
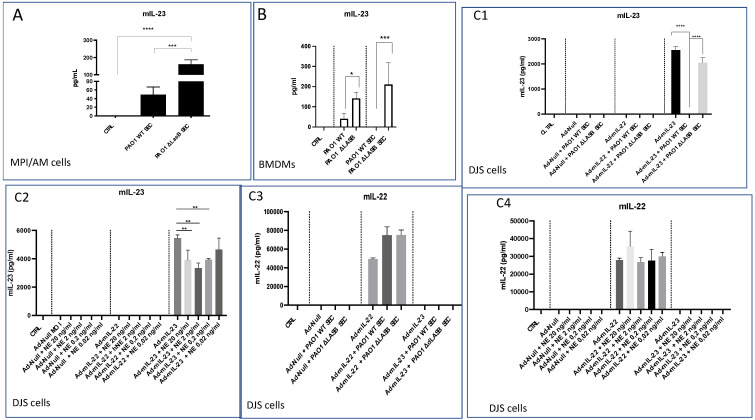
PAO1 LasB and neutrophil elastase down-regulate IL-23, but not IL-22 protein accumulation. (**A**) 0.5.10^6^ MPI cells (in P24 wells) were either mock (serum-free RPMI)-treated or incubated with either WT-PAO1- or ∆LasB-PAO1 secretomes (SEC, 5%) during 6 h. Cell media were collected, centrifuged, and supernatants were assessed by ELISA for IL-23 content. (**B**) Bone-marrow-derived macrophages (BMDMs, seeded at 2.10^6^ cells/P24 plate) were either treated in serum-free RPMI with WT-PAO1- or ∆LasB-PAO1 secretomes (SEC, 1%) or infected with either WT- or ∆LasB-PAO1 (moi 1); 4 h later, cell media were collected, centrifuged, and supernatants were assessed by ELISA for IL-23 content. (**C**) 0.5.10^6^ cells DJS Clara cells (in P24 wells) were infected in serum-free with either Ad-null, Ad-23, or Ad-IL-22 (moi 50); 16 h later, cells were either mock-treated, or treated with WT-PAO1-SEC, ∆LasB-PAO1 SEC (5%, (**C1**–**C3**)), or increasing concentrations of purified neutrophil elastase (NE, (**C2**–**C4**)). After a further 24 h, cell media were collected, centrifuged, and supernatants were assessed by ELISA for IL-23 and IL-22 content. Statistical significance was assessed with ANOVA test, followed by Tukey post hoc multivariate analysis. * = *p* < 0.05. ** = *p* < 0.01; *** = *p* < 0.005; **** = *p* < 0.001.

Whether this was caused directly by neutrophils or *P.a* virulence factors was then investigated. Indeed, we and others have previously shown that virulence factors, including LasB (a metalloprotease secreted by the type 2 secretion system of *P.a*), can target innate immunity [3,4,17,18,19,20,21,22,23]. Firstly, we demonstrated that MPI alveolar macrophages treated with PAO1 WT secretome (SEC) down-regulated IL-23 accumulation in supernatants compared to cells treated with PAO1 ∆LasB SEC (Figure 7A). A similar picture was obtained when BMDMs were treated with either WT or ∆LasB PAO1 SECs or when these cells were infected with live WT or mutant bacteria (Figure 7B). Expectedly, IL-22 was detected neither in MPI macrophages nor in BMDMs (not shown). To further assess whether IL-22 and IL-23 could be targeted by either LasB or NE, we used an artificial adenovirus (Ad)-mediated system of IL-23 and IL-22 over-expression in a Clara epithelial cell line (DJS-2, [24]). By doing so, we confirmed that IL-23 is not only a target of LasB (Figure 7C1) but also of NE since purified NE could significantly decrease IL-23 accumulation in DJS supernatants (Figure 7C2). By contrast, neither LasB nor NE affected IL-22 accumulation (Figure 7C3,C4).

### 2.3. In Vivo Over-Expression of IL-23 Alone Does Not Rescue Mice from a Lethal PAO1 Infection

Because IL-23 can be targeted by both a bacterial product (LasB) and a host inflammatory marker (NE), we hypothesized that over-expression of this cytokine might rescue mice from a lethal dose of PAO1 (10^8^ pfu, a dose that we showed previously in a pilot experiment (not shown) to be equally lethal for WT C57/Bl6, RAG KO and RAG γC double KO). However, we showed that Ad-mediated over-expression of IL-23 alone was not sufficient to improve mice survival (Figure S2).

### 2.4. In Vivo Over-Expression of an IL-23, IL-1β and IL-7 Cocktail Protects against PAO1 Lethal Infection

We then hypothesized that IL-23 activity might be revealed if used in synergy with other cytokines. We, therefore, tested an Ad-mix including IL-23 and IL-1 since we and others showed that IL-1 is not targeted by *P.a* (Figure 3C1, [3,25]) and that it is important in the protection against this bacterium [11,26,27]. In addition, we included IL-7, a lymphocytic ‘homeostatic’ cytokine that has been shown to activate resident lymphocytes with ‘innate immune activities’ [28,29,30].

**Figure 8:**
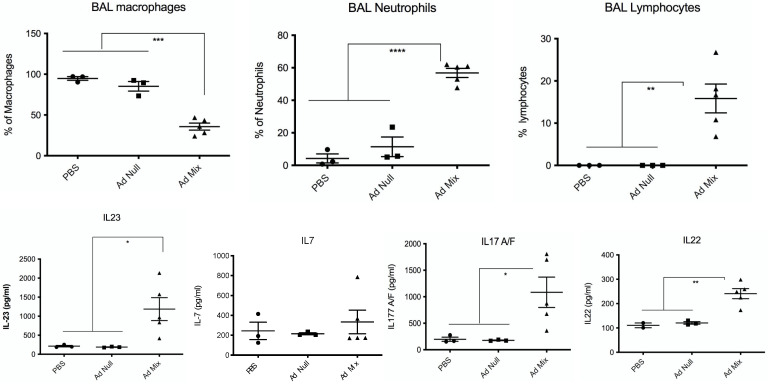
Intra-pulmonary instillation of Adenovirus (Ad)-IL-1β + Ad-IL-23 + Ad-IL-7 up-regulates BAL IL-17, IL-22 accumulation with concomitant BAL neutrophilic and lymphocytic influx. Male C57/Bl6 WT mice were treated intratracheally, through the oro-pharyngeal route at day 0 with either PBS (*n* = 3), with Ad-null (9.10^7^ pfu, *n* = 3) or Ad-mix (3.10^7^ pfu Ad-L1β + 3.10^7^ pfu Ad-IL-23 + 3.10^7^ pfu Ad-IL-7, *n* = 5). Forty-eight hours later, mice were culled, BAL were performed for differential cell analysis (cytospins) and for cytokine measurements (ELISA). Statistical significance was assessed with the ANOVA test, followed by Tukey post hoc multivariate analysis. * *p* < 0.05; ** *p* < 0.01; *** *p* < 0.005; **** *p* < 0.001.

We first validated in WT C57/Bl6 mice this Ad-mix by showing that it could up-regulate BAL IL-23, IL-17 and IL-22 protein levels (Figure 8), and functionally, this treatment induced both a neutrophilic and lymphocytic influx in treated lungs.

**Figure 9:**
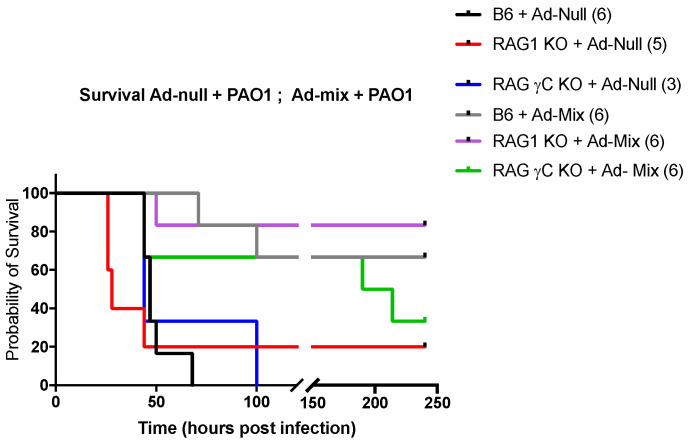
Intra-pulmonary instillation of Adenovirus (Ad)-L1β + Ad-IL-23 + Ad-IL-7 rescues C57/Bl6 WT, RAG KO and RAG γC double KO mice from a lethal PAO1 lung infection. C57/Bl6 WT, RAG KO and RAG γC double KO mice were infected intratracheally, through the oro-pharyngeal route, at day 0 with Ad-null (9.10^7^ pfu) or Ad-mix (3.10^7^ pfu Ad-L1β + 3.10^7^ pfu Ad-IL-23 + 3.10^7^ pfu Ad-IL-7), and 72 h later, mice were infected intra-nasally with 5.10^7^ cfu PAO1 and survival was monitored. Numbers in parenthesis represent the number of mice at the start of the experiment. Survival curves were then plotted using Kaplan–Meier curves. Statistical tests were performed using the Log-rank (Mantel–Cox) tests: C57/Bl6 + Ad-null + PAO1 versus C57/Bl6 + Ad-mix + PAO1: *p* = 0.0005; RAG KO + Ad-null + PAO1 versus RAG KO + Ad-mix + PAO1: *p* = 0.017; RAG γC double KO + Ad-null + PAO1 versus RAG γC double KO + Ad-mix + PAO1: *p* = 0.08.

We then tested in WT C57/Bl6, RAG KO and RAG γc double KOs the potential prophylactic protective effect of Ad-mix against a high dose PAO1, which, as indicated above (not shown), is similarly lethal for C57/Bl6 WT and RAG double KO mice (Figure 9). We showed that this Ad-mix was indeed efficient since all groups receiving the latter survived more than the groups receiving the Ad-null control. Indeed, and somewhat surprisingly, this Ad-mix was efficient in rescuing even RAG KO and RAG γC double KO, therefore making, in this protocol, lymphocytes redundant in the protective process against PAO1 (even though there was a trend for double KO mice surviving less), suggesting instead that lung mucosal stromal and innate myeloid cells (including AMs) may be additional targets of the over-expressed IL-1, IL-23 and IL-7 proteins.

## 3. Discussions

Pseudomonas aeruginosa *(P.a)* is a pathogen causing significant morbidity and mortality, in particular in hospital patients undergoing ventilation and in patients with cystic fibrosis (CF, [1,2]). Indeed, the WHO has recently described *P.a* as one of the most critical pathogens for which new treatment options are urgently required [31]. Although the use of CFTR correctors and potentiators is having a very significant clinical impact in CF patients [32,33], their effect on the long-term resolution of *P.a*-induced lung inflammation and immune responses is still largely unknown. It is therefore important, in the CF context, to pursue mechanistic studies to further understand the basic events underlying lung responses to this bacterium. In that context, it is known that alveolar macrophages (AMs), neutrophils, and epithelial cells are essential in the defense against *P.a* (and broadly speaking, pathogens causing pneumonia, [3,26,27,34,35,36,37]), but less emphasis has been put on the role of the lymphoid lineage compartment, encompassing either ‘classical’ adaptive T cells (TCRαβ, [9,10]), T cells with innate-like activities (TCRγδ, NKT cells, MAIT cells, [11,12,13,14,15,16]), or innate lymphoid cells activated by non-cognate stimuli (NK cells, ILCs, [10,38,39]).

We showed here that both TCR-bearing T cells and innate lymphocytes are important at homeostasis to protect against PAO1 (Figure 3), presumably, as demonstrated by others (e.g., [11,12]), through the production of TCRγδ-derived IL-17 and IL-22 (Figure 4A,B). In an unbiased analysis, C57/Bl6 WT mice infected with a sub-lethal dose of PAO1 demonstrated, expectedly, a lung neutrophilic inflammation, correlating with increased RNA levels of IL-1b, IL-23, IL-6, KC, TNF and Lcn-2 (Figure 4A). In the lungs of both RAG KO and double RAG γc chain KO mice, the main difference with C57/Bl6 WT mice was a reduction in IL-17 and IL-22 protein levels (Figure 4B), associated with a decrease in neutrophilic influx, underlying the role of IL-17 in this process at this time point. Also notable was the increase in PAO1 load in the lungs of double RAG γ chain KO mice, hinting at a potential role of ILCs in controlling this bacterium (Figure 1B). Interestingly, we observed a clear uncoupling between IL-23 and IL-22 RNA and protein levels, suggesting an important post-transcriptional regulation of these specific cytokines following PAO1 infection (Figure 4C). Furthermore, IL-23 and IL-22 protein levels were the only ones inversely correlating with neutrophil influx (Figure 4C) and neutrophil elastase activity (Figure 4D). Relatedly, we demonstrated mechanistically that the ‘IL-23 uncoupling’ was likely caused by both a bacterial product (the metalloprotease LasB) and a host factor (neutrophil elastase) through proteolytic inactivation (Figure 7).

Regarding IL-22, interestingly, Guillon et al. [40] showed that out of the three main serine proteases found in human neutrophils, PR-3 and NE were mostly able to degrade human recombinant IL-22 in vitro, whereas we found that murine IL-22 was resistant to both NE and PAO1 secretomes (Figure 7). The differences can likely be ascribed to species differences and/or the models used (in vitro for Guillon et al. and in cellulo in the present study).

Since IL-23 is a factor probably indispensable for downstream lung IL-22 production [36,37], the ‘IL-22 uncoupling’ demonstrated in vivo here is likely a consequence of IL-23 protein regulation rather than that of IL-22 proteolytic degradation.

Because IL-23 is, with IL-1b, a key developmental/survival cytokine for both ILCs and adaptive γδ T lymphocytes [27,28,29,38,39,41] and has been shown to be essential for the induction of IL-22 in *Klebsiella pneumoniae* and *Streptococcus.pneumoniae* lung infection models [9,38], we hypothesized that over-expression of IL-23, through an adenovirus-mediated strategy would rescue mice against a lethal dose of PAO1. However, the lung overexpression of Ad-IL-23 alone did not confer any protection, neither in RAG KO simple or double KO, nor in C57/Bl6 mice (Figure S2), probably, as suggested previously [11], because of potential ineffective compartmentalization of this cytokine in the luminal alveolar compartment, or the need for co-stimulation with other factors, notably IL-1b.

Indeed, by contrast, the use of an Ad-IL-1b-IL-23-IL-7 mix (IL-7 used as a lymphocyte homeostatic factor able to activate innate T cells production of IL-17 [30]) was able to protect all mice strains (C57/Bl6 WT, RAG KO and double RAG γ chain KO mice), irrespective of the presence of lymphocytes. Although at first sight surprising since IL-23 and IL-7 have been considered to have lymphocytes as main targets, it is likely that in our setup, lung mucosal stromal and innate myeloid cells (including AMs) may be the targets of the over-expressed IL-1, IL-23 and IL-7 proteins. Indeed, the extensive literature shows that stromal cells (epithelial cells and fibroblasts) are established targets for IL-1b, but more interestingly, Sun et al. have also shown that IL-23 promotes antimicrobial pathways in macrophages [42,43]. Similarly, lung epithelial cells have also been shown to be able to produce IL-7 [44], and the latter is also considered a potential adjuvant in the gut [45] and vaginal [46] mucosae.

In conclusion, we demonstrate here that both adaptive and innate lymphocytes are important to control *P.a* infection in a model of acute lung inflammation and that specific post-transcriptional regulation of the IL-23-IL-22 pathway is at play in the lung mucosa following *P.a* infection. Furthermore, we showed that a local administration of an Ad-mediated ‘IL-1b-IL-23-IL-7 mix’ was highly efficacious against *P.a* acute infection (see Figure S3 for a summary of the findings). However, given the well-known activation of the IL-23/IL-17 pathway in autoimmune chronic diseases such as Crohn’s or psoriasis [47,48], further studies will be needed to determine whether a local administration of this ‘cytokine mix’ might be desirable in more chronic lung conditions, such as cystic fibrosis, where lung pathogens such as *P.a* are also prominent.

## 4. Materials and Methods

### 4.1. Recombinant Proteins and ELISA Assays

Murine recombinant proteins (IL-23, IL-22, IL-1b and IL-7) were all from Biolegend (San Diego, CA, USA). ELISAs kits (DuoSet) for IL-22, IL-23, IL-1b, IL-7, IL-17A/F, IL-6, KC, TNF-α and Lcn-2 were all from R&D Systems (Minneapolis, MN, USA).

### 4.2. Adenovirus Constructs and Adenovirus Infection

The control Adenovirus (Ad)- Null [49], Ad-IL-1b (a gift from Dr. C. Richards, McMaster University, Hamilton, Canada), Ad-IL-7 (Applied Biological Materials, Richmond, BC, Canada) and Ad-mIL23 [50], constructs are replication-deficient Ad vectors. Bone-marrow-derived macrophages (BMDMs, see below) were washed 3 times with sterile PBS and infected for 6 h with the different Ad constructs with an MOI of 25 in serum-free RPMI-Glutamax. Supernatants from these infected cells were then assessed by ELISA for cytokine output. In vivo, Ad constructs were instilled intratracheally through the oropharyngeal route, as described [4].

### 4.3. Pseudomonas Aeruginosa O1 Strain, Secretome Production, and Bacterial Load Measurement

PAO1 WT (ATCC 15692) and the PAO1∆LasB strains [3] were kept in a freezing medium (50% Luria broth [LB], 50% glycerol) and stored at −80 °C until use. Before infection experiments, the PAO1 strain was grown overnight in LB in a rotating incubator (200 rpm, 37 °C). The bacterial suspension was then diluted in serum- and antibiotic-free RPMI medium and the optical density (OD) was measured at 600 nm every 2 h until the logarithmic growth phase was reached (0.1 < OD < 0.3; an OD of 0.1 is equivalent to a bacterial concentration of 7.7 × 10^7^ colony forming units (CFU)/mL). Bacteria were instilled in the lungs of mice at the desired concentration. At the end of the experiment, the bacterial load was measured in the lungs by qPCR, using the *P.a* oPRL gene as a reference, as described [51] and as also validated previously in our hands [37,52]. For experiments requiring the use of PAO1 secretomes, these were prepared as described in [3].

### 4.4. Cells, Cell Cultures, and Protocols

#### 4.4.1. Primary Alveolar Macrophages

Primary AMs were isolated from WT by BAL as described previously [3]. BALFs were centrifuged (2000 rpm, 10 min, 4 °C), and cell pellets were re-suspended in RPMI-Glutamax (10% fetal calf serum [FCS], 1000 U/mL penicillin, 100 μg/mL streptomycin). Cells were cultured for 16 h (37 °C, 5% CO_2_) in 48-well Corning Costar culture plates (250,000 cells/well) prior to stimulation or infection with PAO1.

#### 4.4.2. Bone Marrow-Derived Macrophage Generation

Bone marrow was extracted from mice femurs, and cells were washed with PBS and centrifuged at 1400 rpm for 7 min (4 °C). Pelleted cells were then resuspended with lysis buffer (Gibco) for 2 min at room temperature to lyse red blood cells. After another wash, cells were seeded in a complete DMEM medium (10% FCS, 1000 U/mL penicillin, 100 μg/mL streptomycin) containing 30 ng/mL of mouse macrophage colony-stimulating factor (M-CSF) (PeproTech). After two successive medium replacements (on day 3 and day 6), macrophages were detached, washed, and re-suspended in cold sterile PBS on day 9 and kept on ice before further use. For in vitro experiments, cells were seeded in 24-well culture plates (500,000 cells/well) in RPMI-Glutamax (10% FCS, 1000 U/mL penicillin, 100 μg/mL streptomycin) for 12 h prior to stimulation or infection.

#### 4.4.3. Cell Lines

mtCC-DJS2, an SV40T antigen immortalized epithelial Clara cell line [24], a kind gift from Dr. DeMayo (Baylor College of Medicine, Houston, TX, USA), were cultured in DMEM-Glutamax (10% FCS, 1000 U/mL penicillin, 100 μg/mL streptomycin). Cells were placed for 12 h (37 °C, 5% CO_2_) in 24-well Corning Costar culture plates (500,000 cells/well) prior to stimulation.

Alveolar macrophages MPI cells [53] were cultured in RPMI-Glutamax (10% FCS, 1000 U/mL penicillin, 100 μg/mL streptomycin) supplemented with 30 ng/mL GM-CSF (PeproTech). Cells were placed for 16 h (37 °C, 5% CO_2_) in 48-well Corning Costar culture plates (250,000 cells/well) prior to stimulation or Ad-infection.

### 4.5. Animals Procedures

Seven- to ten-week-old male C57BL/6 WT were purchased from Janvier Labs (Le Genest-Saint-Isle, France). Rag1 KO were obtained from the CDTA (Orléans, France), and RagγC double KO mice were a gift from Dr. J. di Santo (Institut Pasteur, Paris, France). Animals were kept in a specific pathogen-free facility under 12-h light/dark cycles, with free access to food and water. Procedures were approved by our local ethical committee and by the French Ministry of Education and Research (agreement number 04537.03). Mice were anesthetized with an intraperitoneal injection of 100 μL of ketamine 500 and xylazine 2% in 0.9% NaCl (10:10:80). They were then treated with Ad-constructs (Ad-null, Ad-IL-1b, Ad-IL-7, Ad-IL-23) prior to PAO1 infection, or only infected with PAO1, depending on the experiments. Instillations were performed intranasally or intratracheally (through the oropharynx) with an air-filled syringe (500 μL), as described previously [3,4]. At the end of the experiment, mice were then euthanized with pentobarbital, tracheae were cannulated, and a BAL (2 mL of total volume) was performed. BALF supernatant was kept at −80 °C until further use, e.g., for cytokine/chemokine measurement, with DuoSet enzyme-linked immunosorbent assay (ELISA) kits (R&D Systems). For neutrophil elastase (NE) activity, BAL fluid samples (diluted in Tris 50 mM; NaCl 0.5 M; Triton X-100; 0.1%; pH 8.0) were incubated at room temperature in the presence of 0.1 mg/mL NE substrate (Methoxysuccinyl-Ala-Ala-Pro-Val-7-amido-4-methylcoumarin, Sigma, excitation and emission wavelength being 460 and 370 nm, respectively), and fluorescence was read over a 3 h period in a TECAN microplate reader. In parallel, the BAL cell pellet was resuspended in 400 μL of PBS for cell type analysis using cytospin centrifugation and Diff-Quik staining (Medion, Diagnostics, Plaisir, France). In parallel, mice lungs were recovered in 1 mL of PBS and homogenized with a FastPrep-24 (MP Biomedicals, Illkirch, France) during two cycles (speed 6, 45 s). Homogenates were then used for cytokine/chemokine measurements or FACS analysis (see below).

### 4.6. Flow Cytometry Analysis

PBS-perfused mice lungs were cut into small pieces and digested with collagenase (1mg/mL) and DNAse (0.01%) (Sigma, Saint-Quentin Fallavier, France) in RPMI media for 30 min at 37 °C under agitation. Lung homogenates were filtered through a 100μm cell strainer. After centrifugation, the pellet was resuspended in 1–2 mL of lysis buffer (ACK lysing buffer, Gibco, Dardilly, France). After further centrifugation and filtration of the resuspended pellet, cells were counted and submitted to FACS analysis. Briefly, cells were first incubated with a cocktail of a viability dye and Fc Block antibody (CD16/32 antibody, 15 min, 4 °C), then washed with FACS buffer (PBS-2% FCS) and incubated (30 min, 4 °C) with a cocktail of cell surface conjugated antibodies (see Supplementary Table S1). For intracellular staining, cells were permeabilized with a mix of GolgiPlug (Brefeldin A, 1/1000) and GolgiStop (Monensin, 1/1500) and incubated at 37 °C for 2 h. After washing, cells were treated as above with specific antibodies (see Supplementary Table S1). Data were acquired the same day with an LSR Fortessa cytometer (BD Biosciences) with BD FACSDiva software and analyzed with FlowJo (Tree Star, Ashland, OR, USA).

### 4.7. RNA Extraction, Reverse Transcription, and qPCR

RNA isolation from cells or tissues was performed with the PureLink RNA Mini Kit (12183018A, Ambion, Life Technologies, Asnières sur Seine, France), following the manufacturer’s instructions and as described previously [3]. qPCR primers were m18S: F: 5′-CTTAGAGGGACAAGTGGCG-3′, R: 5′-ACGCTGAGCCAGTCAGTGTA-3′; mTNF: F: 5′-AGCCGATGGGTTGTACCTT-3′, R: 5′-CAGGGTAATGAGTGGGTTGG-3′; mLcn2: F: 5′-CCAGTTCGCCATGGTATTTT-3′, R: 5′-CCAGTTCGCCATGGTATTTT-3′; mS100A9: F: 5′-AAAGGCTGTGGGAAGTAATTAAGA-3′, R: 5′-GCCATTGAGTAAGCCATTCCC-3′; mIL-1 β: F: 5′-ATGCCACCTTTTGACAGTGATG-3′, R: 5′-GCTCTTGTTGATGTGCTGCT-3′; mIL-6: F: 5′-GCACCAAGACCATCCAATTC-3′, R: 5′-ACCACAGTGAGGAATGTCCA-3′; mIL-10: F: 5′-AAGGCAGTGGAGCAGGTGAA-3′, R: 5′-CCAGCAGACTCAATACACAC-3′; mIL-22: F: 5′-TTCCAGCAGCCATACATCGTC-3′, R: 5′-TCGGAACAGTTTCTCCCCG-3′; mIL-23: F: 5′-AATCTCTGCATGCTAGCCTGG-3′, R: 5′-GATTCATATGTCCCGCTGGTG-3′; KC: F: 5′-GCTGGGATTCACCTCAAGAA-3′, R: 5′-TCTCCGTTACTTGGGGACAC-3′; mIL-17A: F: GCTCCAGAAGGCCCTCAGA, R: 5′- CTTTCCCTCCGCATTGACA-3′.

### 4.8. Statistical Analysis

Data were analyzed with GraphPad Prism Software 9.0.2. Statistical analysis was performed with either a non-parametric test (Kruskal–Wallis and Dunn’s posttest) or one-way or two-way ANOVA followed by the appropriate multi-comparison post hoc Tukey’s test. Survival curves in murine model experiments were plotted with Kaplan–Meier curves, and statistical testing was performed with the log-rank (Mantel–Cox) test. PCA and correlation matrix graphs were generated with the same software. Differences were considered statistically significant when *p* was < 0.05 and are labeled as follows: * *p* < 0.05; ** *p* < 0.01; *** *p* < 0.001; **** *p* < 0.0001.

## Data Availability

All the original data can be provided, upon reasonable request.

## Supplementary Materials

The following supporting information can be downloaded at: https://www.mdpi.com/article/10.3390/ijms23158427/s1.
