# Supplementary material for: Pseudomonas Aeruginosa Lung Infection Subverts Lymphocytic Responses through IL-23 and IL-22 Post-Transcriptional Regulation
Source: Int J Mol Sci. 2022 Jul 29;23(15):8427. doi: 10.3390/ijms23158427 (PMC9369422; doi:10.3390/ijms23158427)
Supplement: Supplementary file 1 [file ijms-23-08427-s001.zip › Supplemental Table and Figures Legends (1).pdf]

Supplemental Table and Figures Legends

Supplemental Table :

The different antibodies used in the FACS are listed, with the fluorochrome and the dilutions used.

Fig S1 : Gating strategy for the detection of lung Tγδ and ILC cells, following PAO1 lung infection

A)Tγδ cells : Following lung homogenization (see Material and Methods), singlets from a FSC/SSC lymphocytic gate were first selected, then gated for CD45+/viability, followed by Tγδ/CD3 gating, and finally analysed for IL-17A and IL-22 expression by intra-cellular staining.

B)ILC cells : Following lung homogenization (see Material and Methods), singlets from a FSC/SSC lymphocytic gate were first selected, then gated for CD45+/viability, followed by CD3/CD5 gating. Lin- CD3-CD5- were then selected, followed by a CD90/CD127 selection. CD90+ cells (ILCs) were then sub-divided into ILC3 cells (ST2/RORγt) or into IL-17/IL-22 ILC-expressing cells by intra-cellular staining.

Fig S2 : Intra-pulmonary instillation of Ad-IL-23 alone does not rescue C57/Bl6 WT, RAG KO and RAG γC double KO mice from a lethal PAO1 lung infection

C57/Bl6 WT, RAG KO and RAG γC double KO mice were infected intra-tracheally, through the oro-pharyngeal route, at day 0 with Ad-null (3.10^7 pfu) or Ad-IL-23 (3.10^7 pfu). 72 hours later, mice were infected intra-nasally with 5.10^7 cfu PAO1 and survival was monitored. Numbers in parenthesis represent the number of mice at the start of the experiment. Survival curves were then plotted using Kaplan-Meier curves. Statistical tests were performed using the Log-rank (Mantel-Cox) tests (no statistical significance).
