# Supplementary material for: Pseudomonas Aeruginosa Lung Infection Subverts Lymphocytic Responses through IL-23 and IL-22 Post-Transcriptional Regulation
Source: Int J Mol Sci. 2022 Jul 29;23(15):8427. doi: 10.3390/ijms23158427 (PMC9369422; doi:10.3390/ijms23158427)
Supplement: Supplementary file 1 [file ijms-23-08427-s001.zip › Table Supplemental.pdf]

Table Supplemental

FACS antibodies :

Antibody	Fluorochrome	Dilution
Viablility Dye	Amcyan	1/500
CD16/CD32		1/100

ILC mix

Membrane

CD45	PB	1/100
CD3	AF100	1/100
CD5	PE	1/400
NKP46	efluor660(APC)	1/50
NK1.1	APC Cy7	1/100
CD127	PE-Cy7	1/50
CD90.2	BV605 (=Q dot 605)	1/300
ST2	FITC	1/50

Intracellular

IL22	Percpcy eFluor710 (=percp)	1/50
IL17A	BV711	1/50
RoRgt	PC-CF594 (=PE Texas Red)	1/50

iNKT mix

Membrane

CD45	PECY7	1/400

TCRb	FITC	1/100
TT	PE	1/1000
CD69	AF700	1/100

Intracellular

RORgt	PE-CF594	1/50
IL22	PercpeFluor710	1/50
IL17A	BV711	1/50

Tγδ mix

Membrane

CD45	PECY7	1/400
CD3	FITC	1/100
TCRgd	Percp	1/200
CD69	AF700	1/100

Intracellular

IL22	PE	1/50
IL17A	APC	1/50
