# Supplementary figures and images for: Pseudomonas Aeruginosa Lung Infection Subverts Lymphocytic Responses through IL-23 and IL-22 Post-Transcriptional Regulation
Source: Int J Mol Sci. 2022 Jul 29;23(15):8427. doi: 10.3390/ijms23158427 (PMC9369422; doi:10.3390/ijms23158427)

$T\gamma\delta$

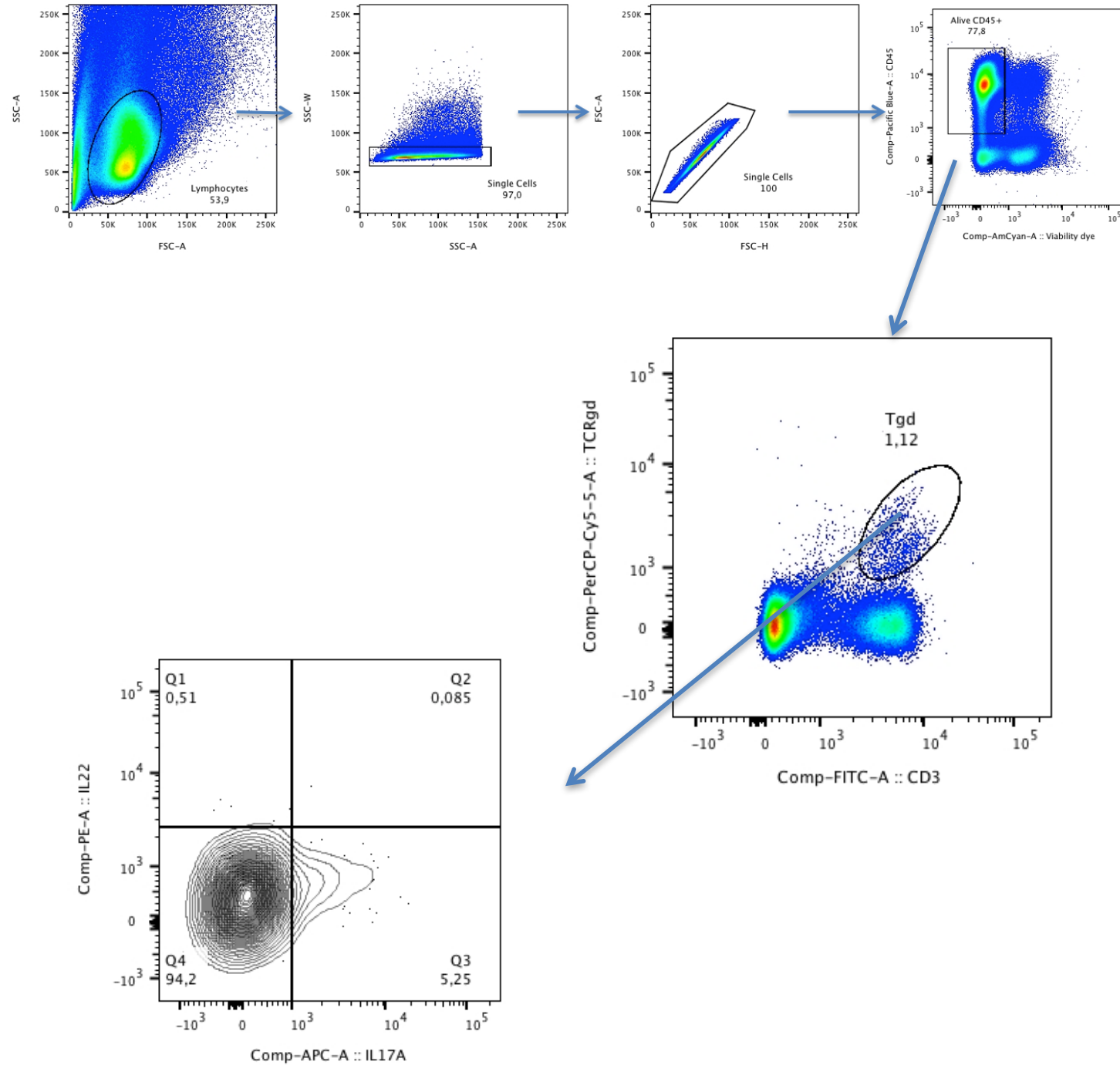

Fig S1A

# ILCs

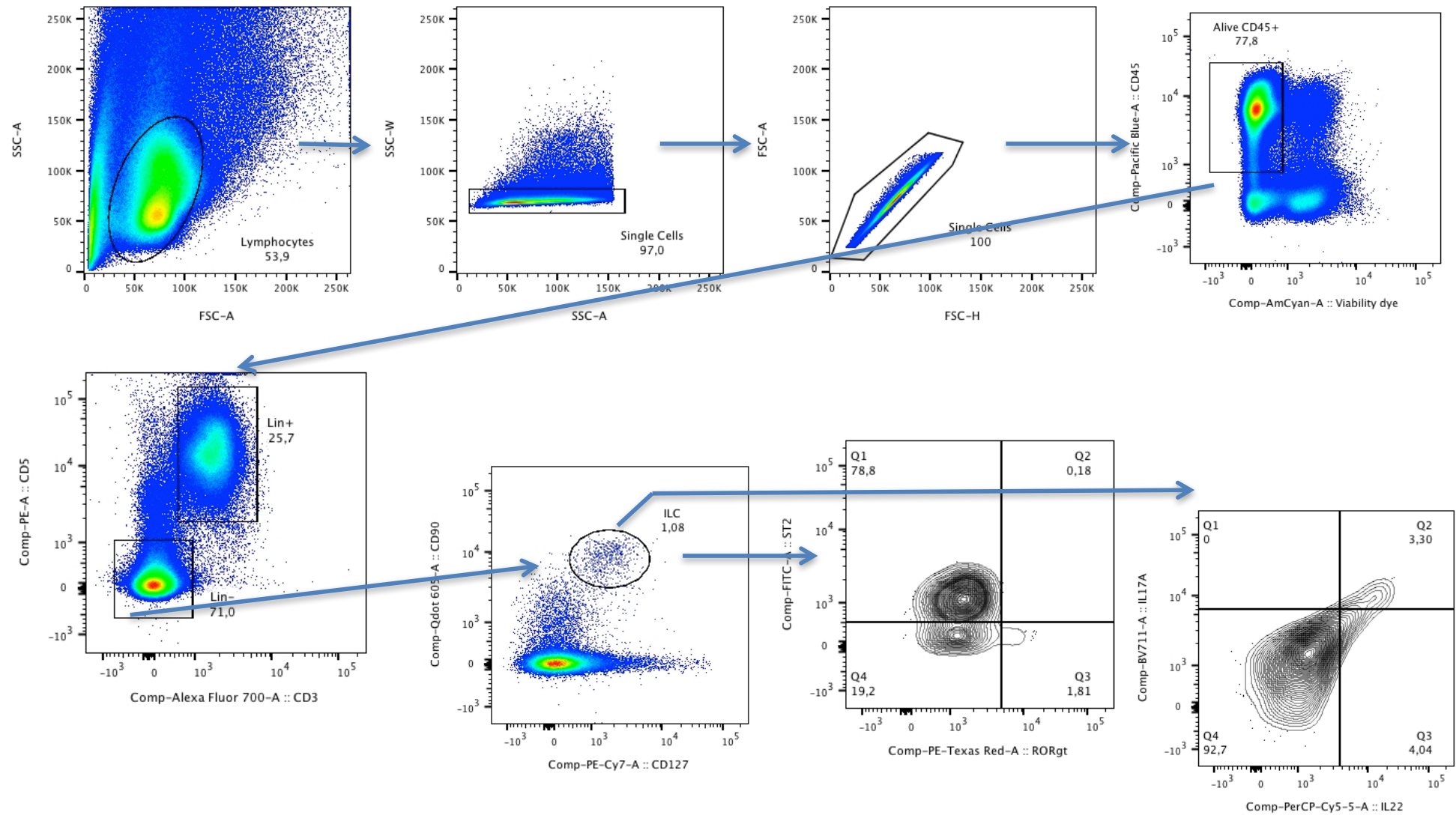

Fig S1-B

Supplement: Supplementary file 1 [file ijms-23-08427-s001.zip › Fig S1.pdf]

### Survival Ad-null + PAO1 ; Ad-IL-23 + PAO1

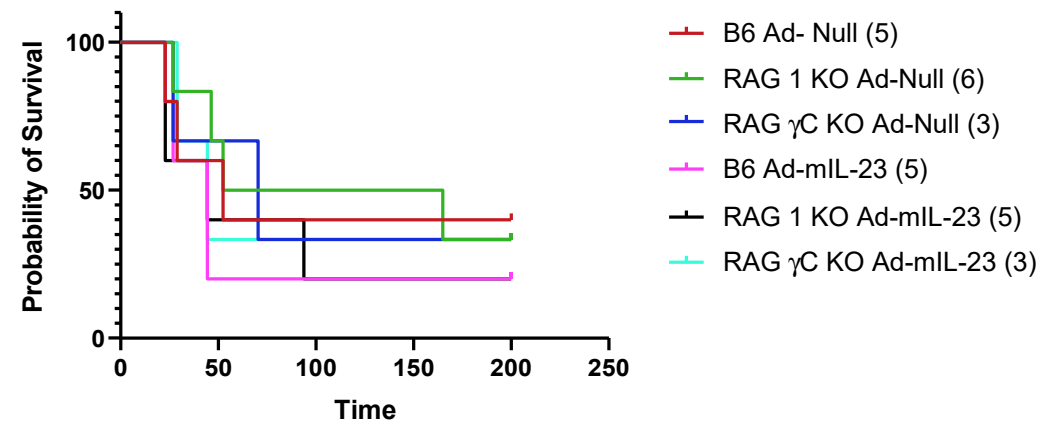

Fig S2

Supplement: Supplementary file 1 [file ijms-23-08427-s001.zip › Fig S2.pdf]
